# Supplementary material for: Redox dynamics and surface structures of an active palladium catalyst during methane oxidation
Source: Nat Commun. 2024 Jun 1;15:4678. doi: 10.1038/s41467-024-49134-y (PMC11144237; doi:10.1038/s41467-024-49134-y)
Supplement: Supplementary file 1 — Supplementary Information [file 41467_2024_49134_MOESM1_ESM.pdf]

Supplementary Information

# Redox Dynamics and Surface Structures of an Active Palladium Catalyst during Methane Oxidation

Shengnan Yue,[1,2,¶] Praveen C. S.,[3,¶] Alexander Klyushin,[4,¶] Alexey Fedorov,[5] Masahiro Hashimoto,[6] Qian Li,[1,2] Travis Jones,[7]* Panpan Liu,[1,2] Wenqian Yu,[1,2] Marc-Georg Willinger,[8,9] and Xing Huang[1,2,8]*

[1] College of Chemistry, Fuzhou University, 350108 Fuzhou, China

[2] Qingyuan Innovation Laboratory, 362801 Quanzhou, China

[3] International School of Photonics, Cochin University of Science and Technology, 682022 Cochin, India

[4] MAX IV Laboratory, Lund University, Box 118, Lund, 22100, Sweden

[5] Department of Mechanical and Process Engineering, ETH Zurich, 8092 Zurich, Switzerland

[6] JEOL (EUROPE) SAS, allée de Giverny, 78290 Croissy-sur-Seine, France

[7] Theoretical Division, Los Alamos National Laboratory, 87545 New Mexico, USA

[8] Scientific Center for Optical and Electron Microscopy, ETH Zurich, 8093 Zurich, Switzerland

[9] Department of Chemistry, Technical University of Munich, 85748 Garching, Germany

[¶] Equal contribution

Emails: tejones@lanl.gov; xinghuang@fzu.edu.cn

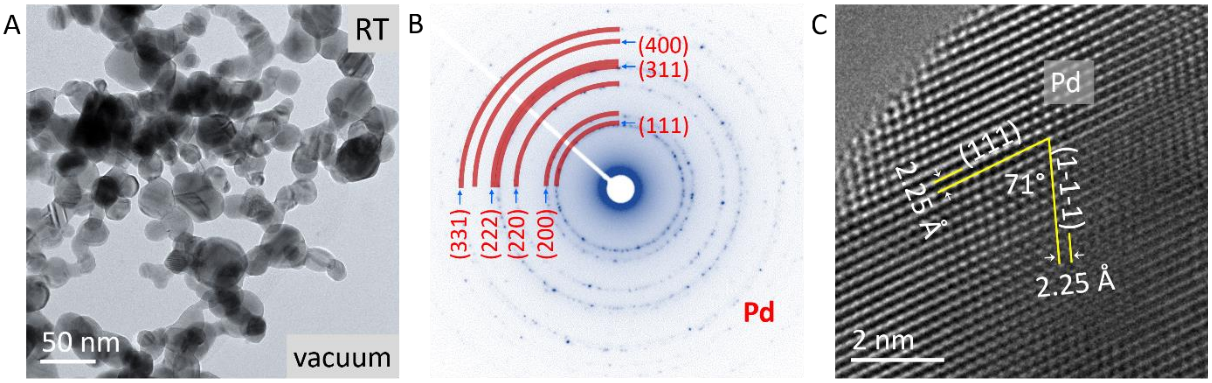


**Figure S1.** (A) TEM image, (B) SAED patterns and (C) HRTEM image of the initial metallic Pd NPs recorded at room temperature.

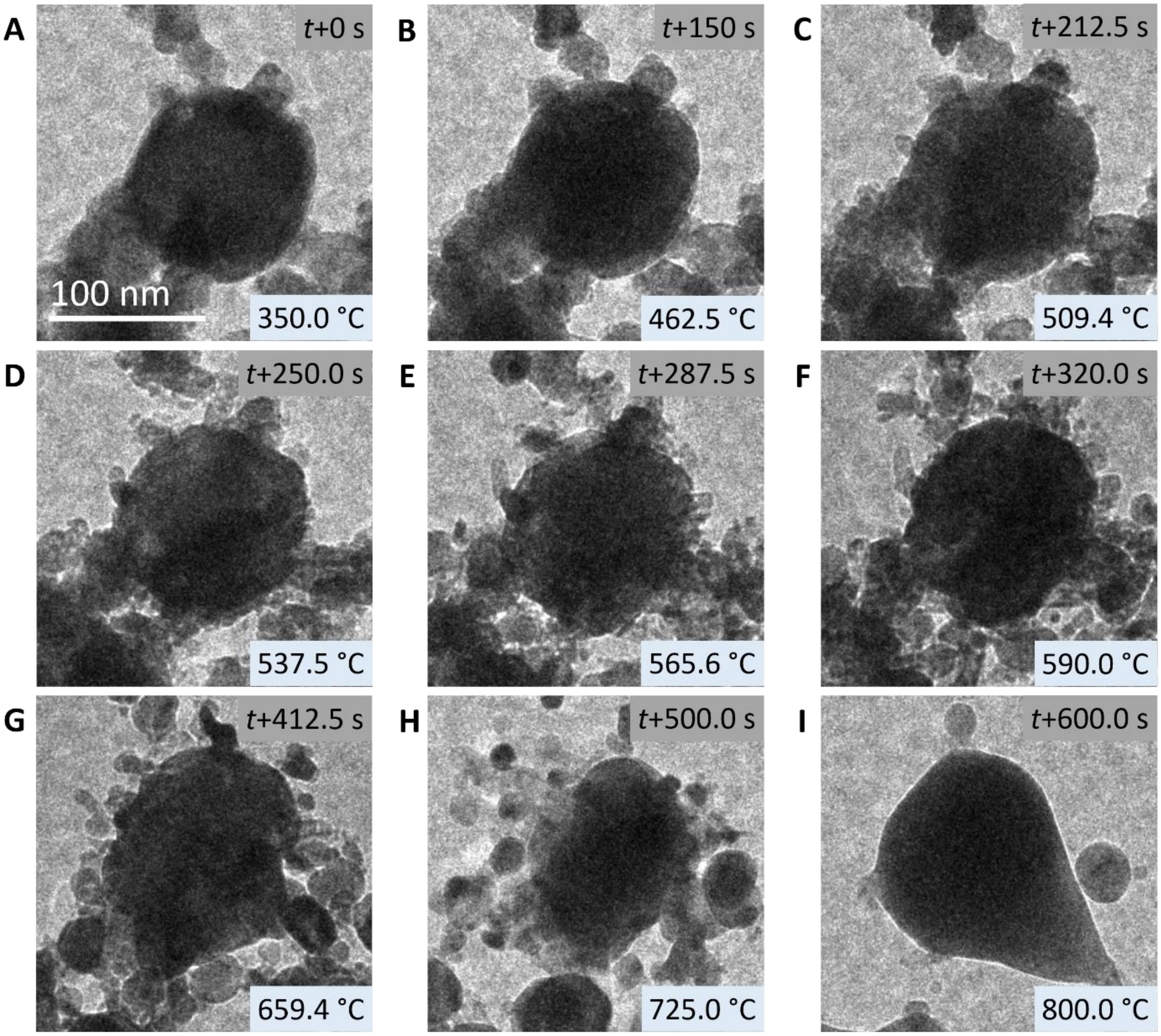


**Figure S2.** (A-I) *In situ* observation of morphological transformation of Pd catalyst during increasing temperature from 350 to 800 °C in reactive atmosphere ($p(CH_4)$=39.5 mbar, $p(O_2)$=8.8 mbar and $p$(He)=131.7 mbar).

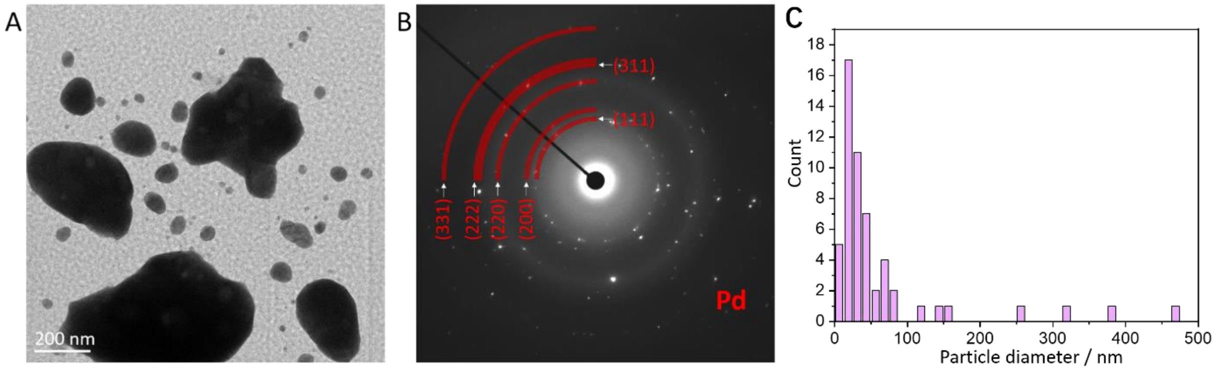


**Figure S3.** *In situ* (A) TEM image and corresponding (B) SAED patterns of Pd particles at 800 °C ($p(CH_4)$=39.5 mbar, $p(O_2)$=8.8 mbar and $p$(He)=131.7 mbar). (C) Particle size distribution. Upon heating to 800 °C, the majority of Pd nanoparticles sintered into larger particles measuring up to 500 nm, while relatively smaller particles ranging from 10-50 nm continued to co-exist. This sintering process led to a notable decrease in the countable number of particles.

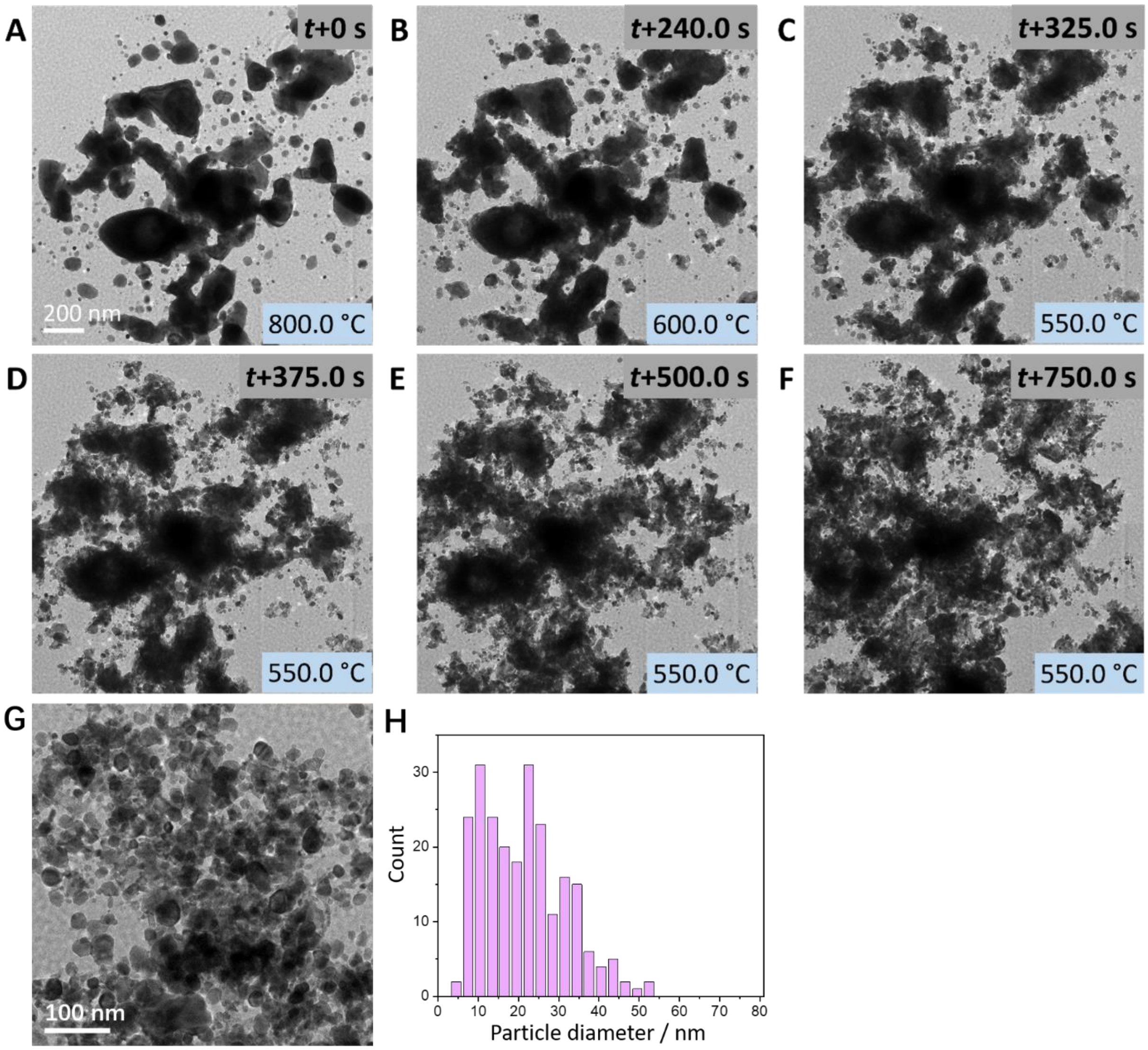


**Figure S4.** (A-F) *In situ* observation of particle fragmentation during temperature decrease from 800 to 550 °C ($p(CH_4)$=38.6 mbar, $p(O_2)$=12.9 mbar and $p$(He)=128.5 mbar) at full scale. (G) TEM image of Pd particles after reaching a dynamic equilibrium state at the applied condition and (H) particle size distribution.

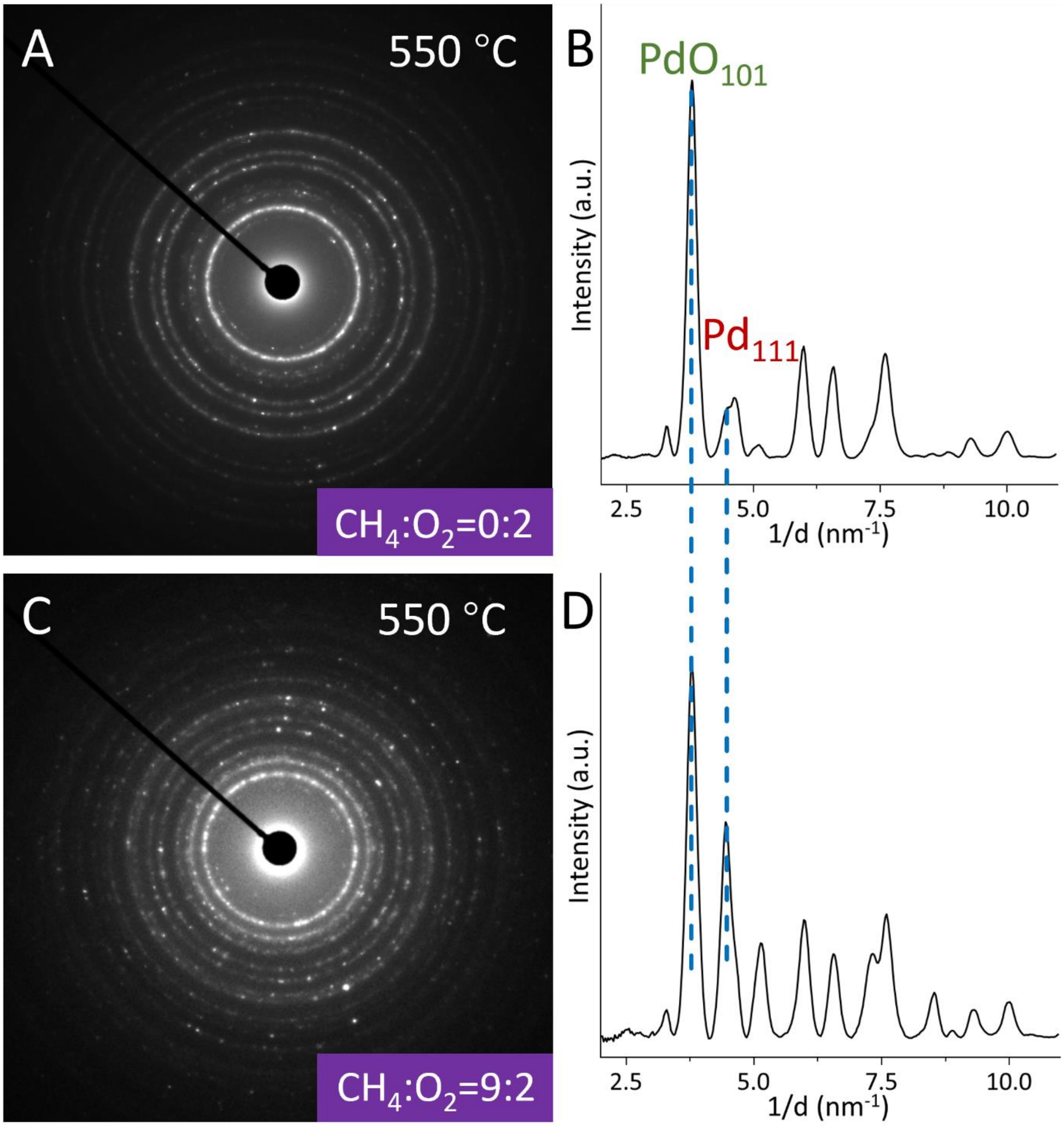


**Figure S5.** *In situ* SAED patterns and the derived line profiles at 550 °C in (A,B) $O_2$/He ($p(CH_4)$=30 mbar, $p$(He)=150 mbar) and (C,D) $CH_4/O_2$/He mixture ($p(CH_4)$=39.5 mbar, $p(O_2)$=8.8 mbar and $p$(He)=131.7 mbar).

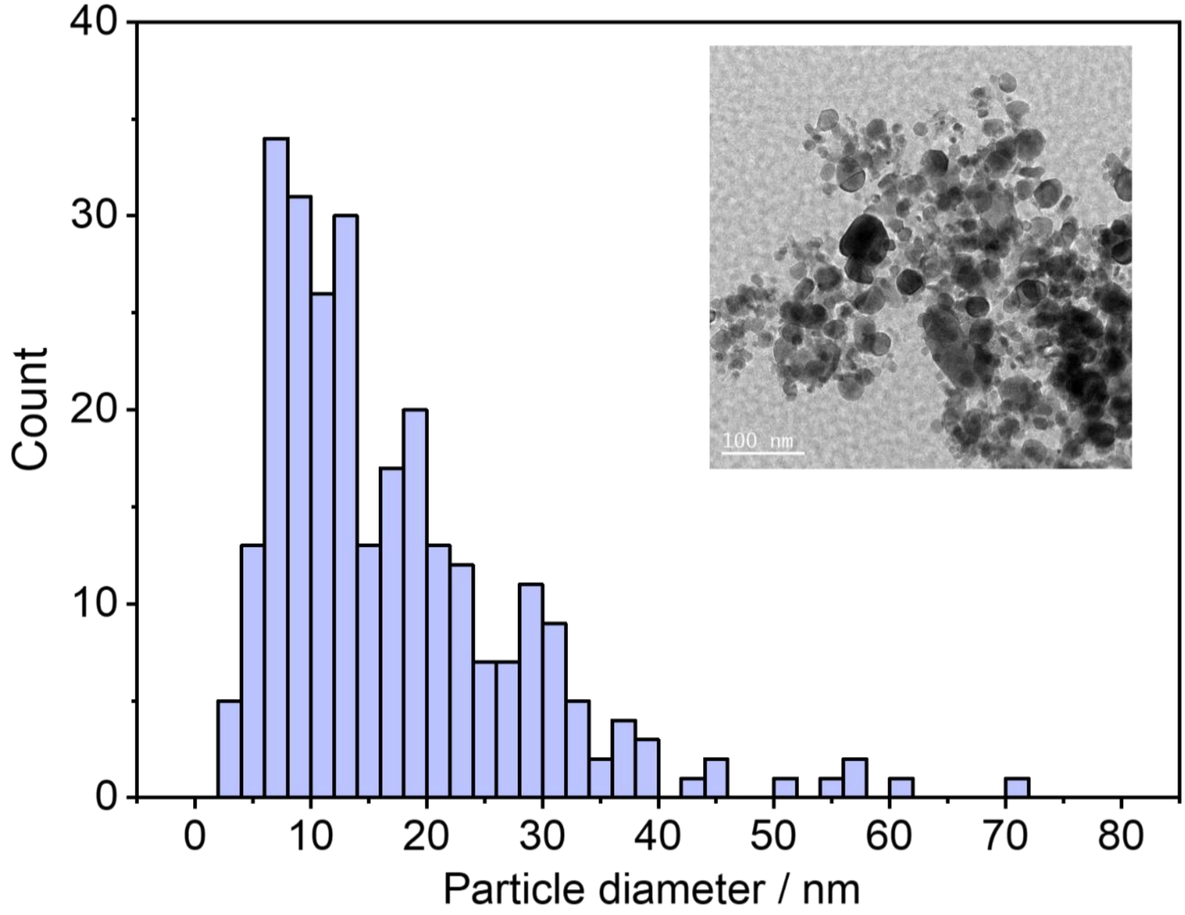


**Figure S6.** *In situ* TEM image (inset) and particle size distribution at 550 °C ($p$($CH_4$)=39.5 mbar, $p$($O_2$)=8.8 mbar and $p$(He)=131.7 mbar).

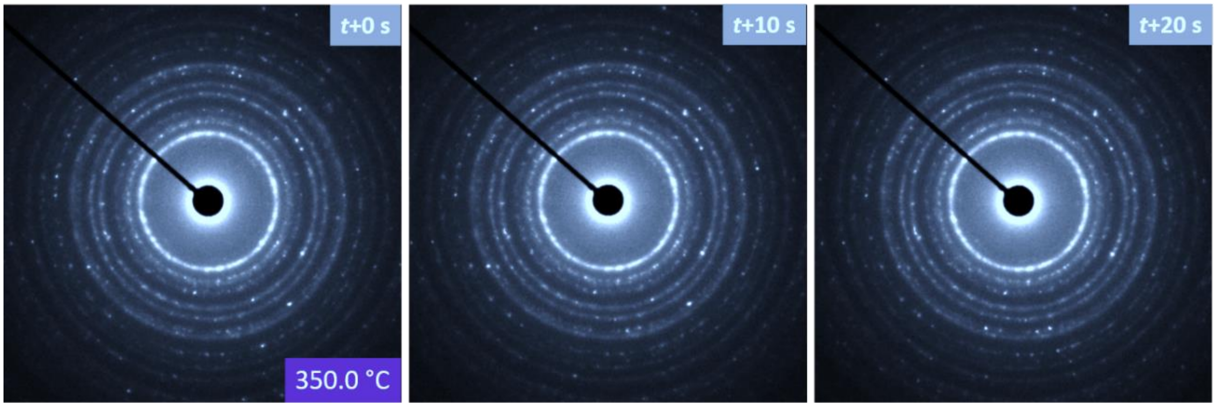


**Figure S7.** *In situ* SAED data recorded at 350 °C ($p(CH_4)$=39.5 mbar, $p(O_2)$=8.8 mbar and $p$(He)=131.7 mbar), which barely change with time due to absence of phase oscillations.

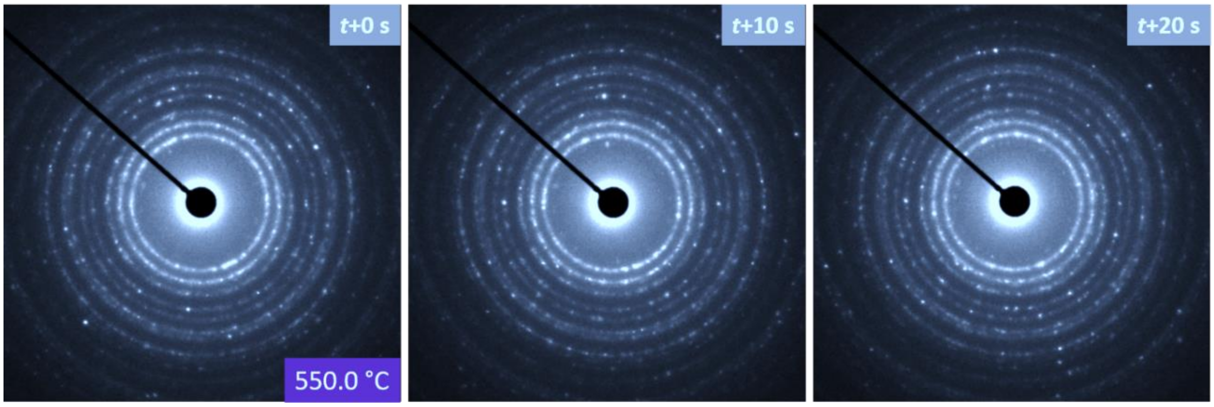


**Figure S8.** *In situ* SAED data recorded at 550 °C ($p(CH_4)$=39.5 mbar, $p(O_2)$=8.8 mbar and $p$(He)=131.7 mbar), which change dynamically due to phase oscillations.

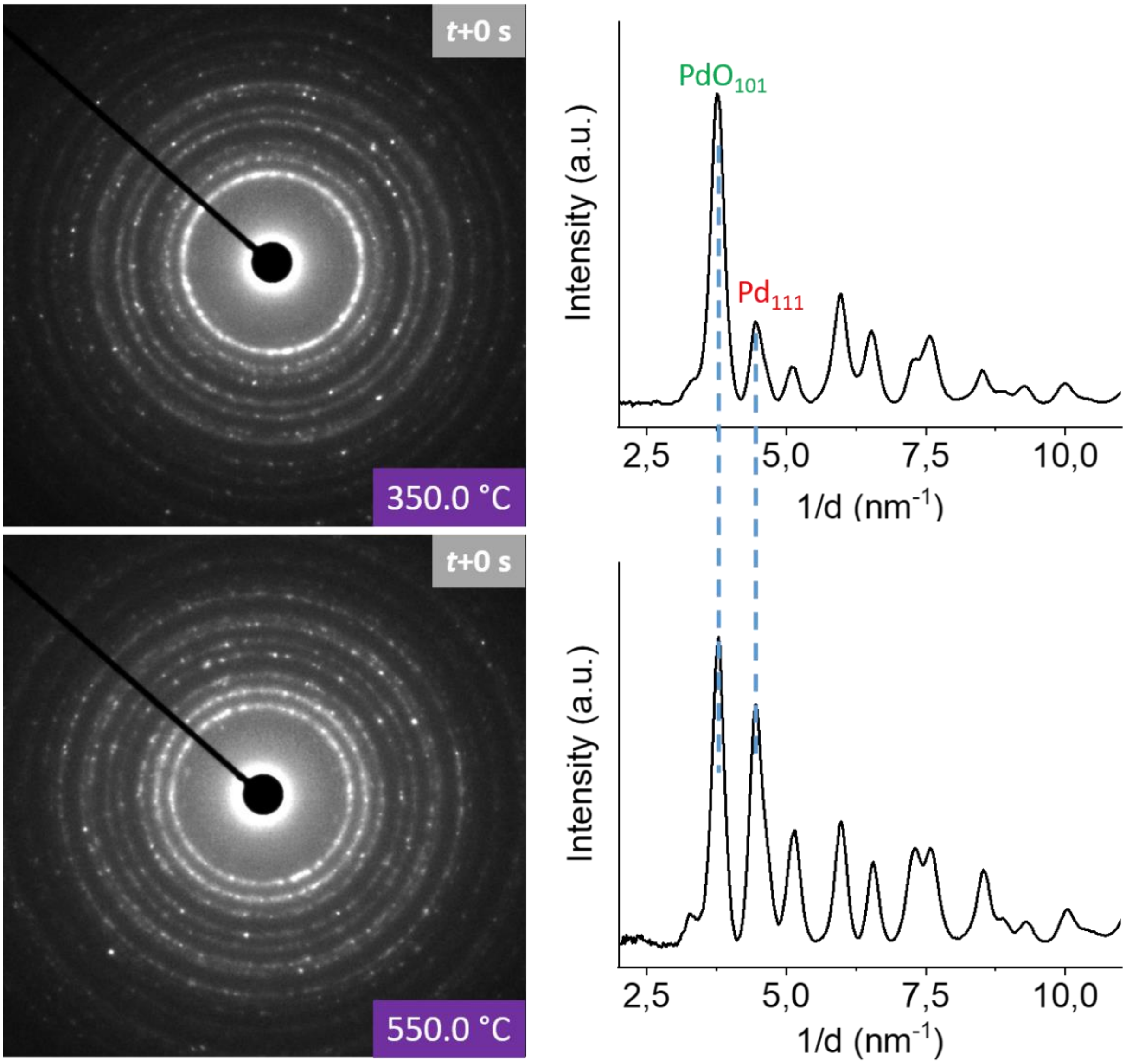


**Figure S9.** *In situ* SAED patterns and the derived line profiles at 350 (top panel) and 550 °C (bottom panel) ($p(CH_4)$=39.5 mbar, $p(O_2)$=8.8 mbar and $p$(He)=131.7 mbar), showing an increased fraction of metallic Pd at 550 °C.

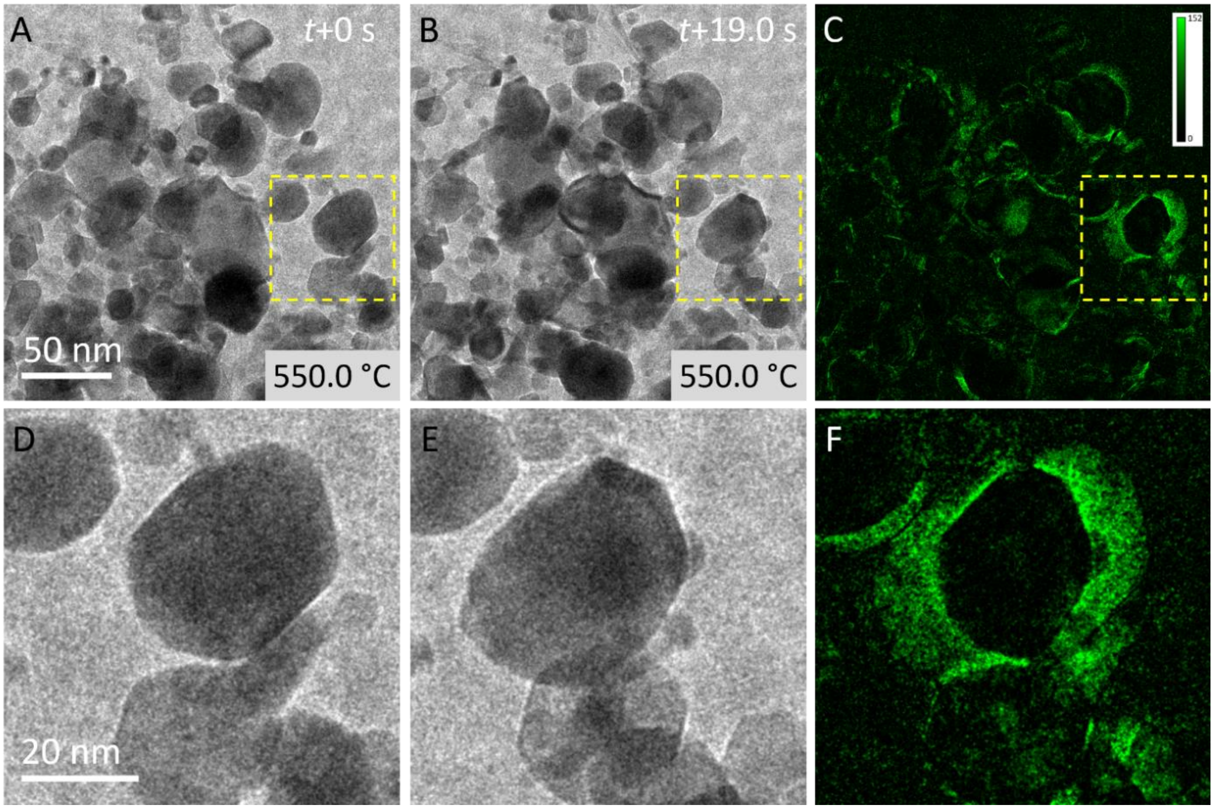


**Figure S10.** (A,B) *In situ* observation at 550 °C without beam irradiation between the two shots ($p(CH_4)$=39.5 mbar, $p(O_2)$=8.8 mbar and $p$(He)=131.7 mbar). (C) Difference between images A and B**.** (D-F) Enlarged images from the dotted squares indicated in (A-C). Dose rate was 3800 e $nm^{-2}$ $s^{-1}$.

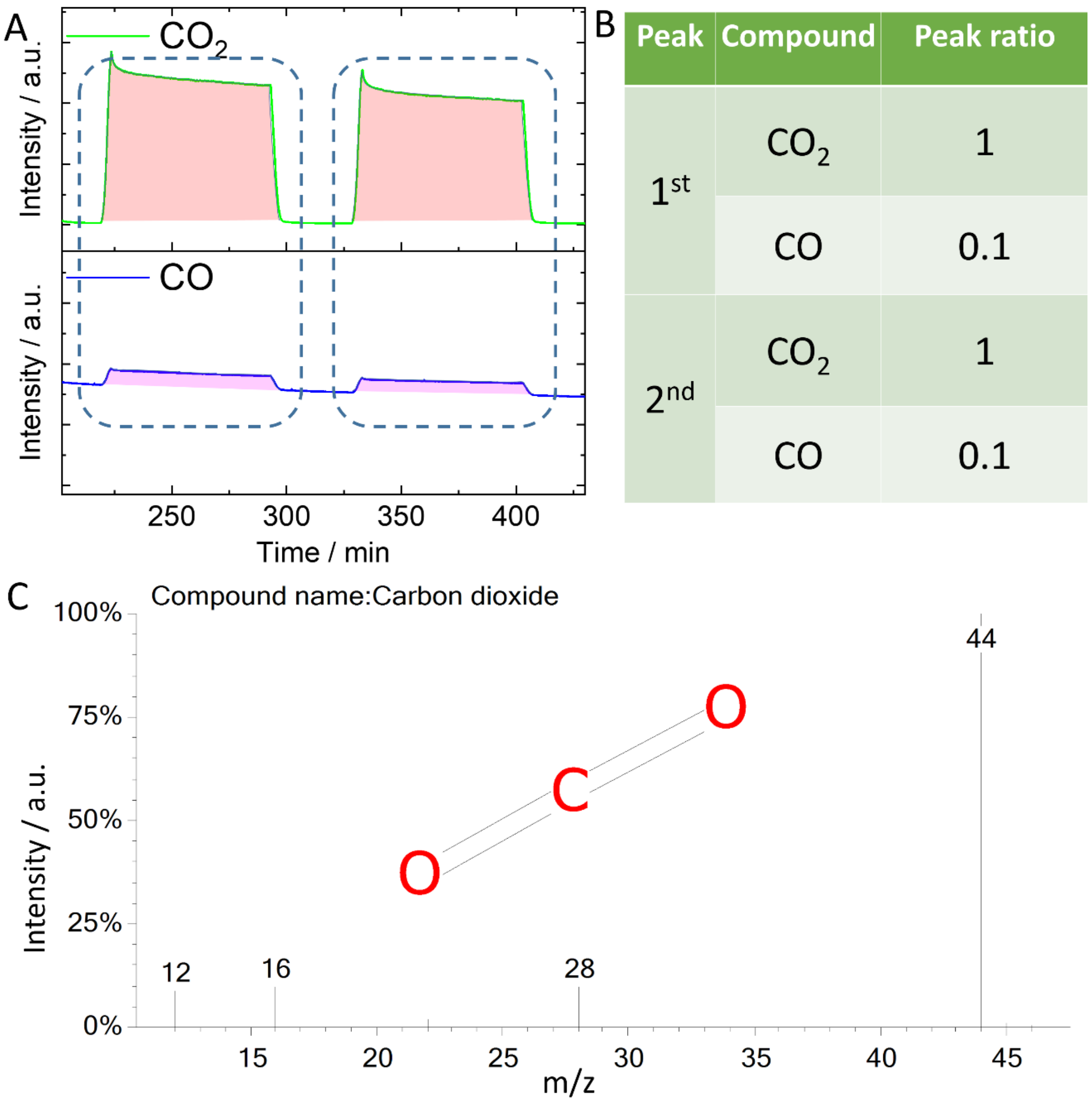


**Figure S11.** (A) MS data ($CO_2$ and CO) recorded during *in situ* experiments (*p*($CH_4$)=39.5 mbar, *p*($O_2$)=8.8 mbar and *p*(He)=131.7 mbar). (B) Peak ratio of $CO_2$ and CO. (C) $CO_2$ mass spectrum from NIST library.(*1*)

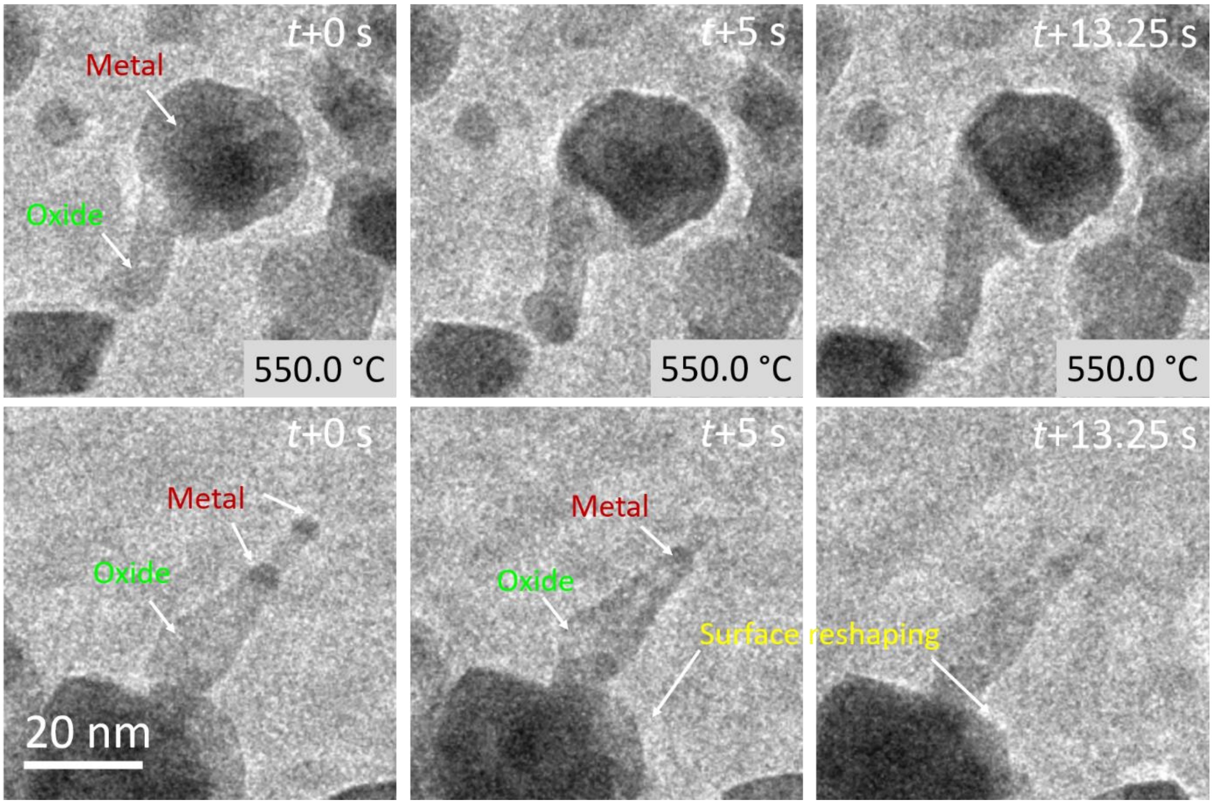


**Figure S12.** *In situ* observations recorded at 550 °C in the reaction mixture ($p(CH_4)$=38.6 mbar, $p(O_2)$=12.9 mbar and $p$(He)=128.5 mbar) at low magnifications, showing phase oscillation-induced structural dynamics (reshaping, outgrowth, and etc.), which are similar to these recorded at higher magnifications. Dose rate is 2300 e nm$^{-2}$ s$^{-1}$.

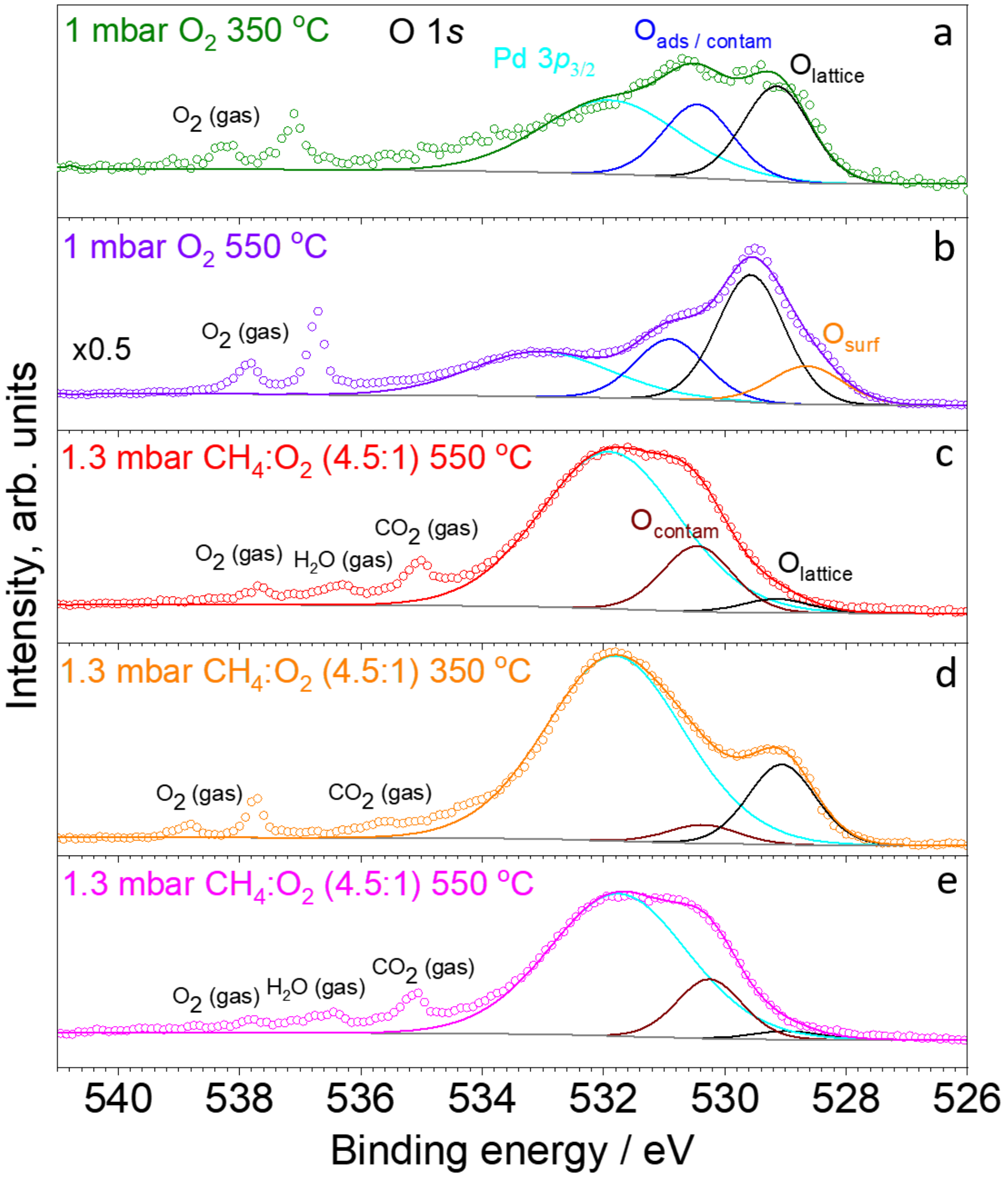


**Figure S13.** O 1*s* XPS data of Pd NPs in (a) 1 mbar $O_2$ at 350 °C, (b) 1 mbar $O_2$ at 550 °C, (c) 1.3 mbar and $CH_4/O_2$ = 4.5:1 at 550 °C, (d) 1.3 mbar $CH_4/O_2$ = 4.5:1 at 350 °C, and (e) 1.3 mbar $CH_4/O_2$ = 4.5:1 at 550 °C after temperature decrease.

**Table S1.** The XPS fitting parameters of Pd 3$d_{5/2}$ spectra.

| | $Pd^0$ | | | | $Pd^{\delta+}$ | | | | $Pd^{2+}$ | | | |
|---|---|---|---|---|---|---|---|---|---|---|---|---|
| | Shape | BE, eV | FWHM, eV | % | Shape | BE, eV | FWHM, eV | % | Shape | BE, eV | FWHM, eV | % |
| $O_2$, 350 °C | DS(0.12,400) | 335.0 | 0.7 | 51.3 | GL(80) | 335.5 | 0.7 | 3.0 | GL(80) | 336.3 | 0.8 | 45.7 |
| $O_2$, 550 °C | DS(0.12,400) | - | - | - | - | - | - | - | GL(80) | 336.4 | 0.8 | 100 |
| $CH_4$:$O_2$, 550 °C | DS(0.12,400) | 334.9 | 0.6 | 79.6 | GL(80) | 335.3 | 0.8 | 19.9 | GL(80) | 336.1 | 0.8 | 0.5 |
| $CH_4$:$O_2$, 350 °C | DS(0.12,400) | 334.9 | 0.5 | 61.0 | GL(80) | 335.3 | 0.7 | 30.9 | GL(80) | 336.1 | 0.8 | 8.1 |
| $CH_4$:$O_2$, 550 °C_2 | DS(0.12,400) | 334.8 | 0.6 | 81.4 | GL(80) | 335.3 | 0.8 | 18.6 | - | - | - | - |

**Table S2.** The XPS fitting parameters of O 1*s* spectra.

| | Pd 3$p_{3/2}$ | | | | O contamination | | | | O lattice | | | | O surface | | | |
|---|---|---|---|---|---|---|---|---|---|---|---|---|---|---|---|---|
| | Shape | BE, eV | FWHM, eV | % | Shape | BE, eV | FWHM, eV | % | Shape | BE, eV | FWHM, eV | % | Shape | BE, eV | FWHM, eV | % |
| $O_2$, 350 °C | GL(30) | 531.9 | 2.7 | 47.0 | GL(30) | 530.4 | 1.3 | 23.3 | GL(30) | 529.1 | 1.3 | 29.7 | - | - | - | - |
| $O_2$, 550 °C | GL(30) | 533.0 | 2.7 | 28.7 | GL(30) | 530.9 | 1.4 | 19.3 | GL(30) | 529.6 | 1.3 | 39.3 | GL(30) | 528.7 | 1.4 | 12.7 |
| $CH_4$:$O_2$, 550 °C | GL(30) | 531.9 | 2.7 | 79.7 | GL(30) | 530.5 | 1.4 | 17.0 | GL(30) | 529.2 | 1.3 | 3.3 | - | - | - | - |
| $CH_4$:$O_2$, 350 °C | GL(30) | 531.8 | 2.7 | 79.3 | GL(30) | 530.4 | 1.4 | 4.0 | GL(30) | 529.1 | 1.3 | 16.7 | - | - | - | - |
| $CH_4$:$O_2$, 550 °C_2 | GL(30) | 531.8 | 2.7 | 81.6 | GL(30) | 530.3 | 1.3 | 16.0 | GL(30) | 529.0 | 1.3 | 2.4 | - | - | - | - |

**Supplementary Note One: Additional discussion on XPS data:**

We employed DFT to compute the Pd 3$d_{5/2}$ and O 1$s$ core level binding energies using the ΔSCF method. Oxygen in bulk PdO was used for the O 1$s$ reference state assuming a binding energy of 529.1 eV (Table S2). Metal was used as the Pd 3$d_{5/2}$ reference assuming a value of 334.9 eV (Table S1). For the O 1$s$ calculations we used the O.star1s-pbe-van_gipaw pseudopotential for the excited oxygen atom, and for Pd, we generated a Pd pseudo with a core hole starting from the PSLibrary PAW input file using the ld1.x code of the QE package. The oxygen pseudopotential is available at http://pseudopotentials.quantum-espresso.org/upf_files/O.star1s-pbe-van_gipaw.UPF. Tables S3 and S4 show the results for the Pd 3$d_{5/2}$ and O 1$s$ calculations.

As we can see in Table S3 (and Figure 4), the Pd 3$d_{5/2}$ energy of Pd is 334.9 eV and in PdO it is 335.7 eV, where the 0.8 eV shift indicates the presence of $Pd^{2+}$. Screening different structures within DFT offers insight into the mechanism, where we have focused on Pd atoms in the (i) bulk, (ii) subsurface and (iii) surface of the structures considered in this work. We begin with the pristine Pd(100) surface. In this case, Pd in the bulk and subsurface have a BE of 334.9 eV, with the surface palladium atom showing a surface core level shift of −0.4 eV (334.5 eV) due to the unsaturated bonds at the surface. On the PdO(001) surface, the bulk and subsurface Pd atoms have a BE of 335.7 eV. The BE of the surface Pd atom on PdO(001) is shifted +0.4 eV (336.1 eV) relative to the bulk oxide, showing the surface atoms are oxidized to $Pd^{2+\delta}$ due to the surface oxygen. From our mechanistic calculations we find this highly oxidized species is extremely reactive, which agrees with its absence from the measured spectrum.

We also considered the role of strain on the XPS BEs. First we consider a PdO(001) surface strained to match the Pd(100) surface. In this case, the Pd 3$d_{5/2}$ BE is slightly higher than that of the bulk PdO reference, showing the influence of lattice strain on the BEs. Now, if we look at the BEs of Pd in the PdO(001)/Pd(100) structure, the bulk Pd atom shows the same binding energy (334.9 eV) as the bulk Pd

atom within the pristine Pd(100), corresponding to a $Pd^0$ metallic state, as expected. The surface Pd atoms that are bound to oxygen, however, show much higher BEs (336.6 eV), matching those computed for the strained PdO surface. The subsurface Pd atoms show an intermediate BE of 335.5 eV. This can be ascribed to a partially oxidized metal, or $Pd^{1+\delta}$. This corroborates the experimental XPS, suggesting the presence of partially oxidized Pd at the metal oxide interface. To further test this assignment, we removed the terminating oxygen layer from the PdO(001)/Pd(100) surface and calculated the BEs. In this case, the surface and subsurface Pd atoms show BEs between that of a neutral Pd atom and $Pd^{2+}$. Finally, we estimated the binding energy of Pd atoms close to an oxygen vacancy in the surface of the PdO(001)/Pd(100) structure and observed that the Pd atom close to the vacancy, which is now partially reduced, shows a BE of 335.5 eV, like that of $Pd^{1+\delta}$ (335.4 eV). Therefore, we find that $Pd^{1+\delta}$, may appear near oxygen vacancies in the oxide or at the metal oxide interface.

**Table S3.** Pd $3d_{5/2}$ BE calculated using the ΔSCF method. For all the surfaces considered, we have computed the BE of the bulk Pd atom, a subsurface Pd atom and a surface Pd (top most Pd layer) atom. Bulk Pd metal was taken as the reference state with a Pd $3d_{5/2}$ of 334.9 eV

| **System** | **Pd-atom-Studied** | **Core-level BE (eV)** |
|---|---|---|
| **Pd(Bulk)** | | 334.9 |
| **PdO(Bulk)** | Pd(Bulk) | 335.7 |
| **Pd(100)Surface** | Pd(Bulk) | 334.9 |
| | Pd(Sub-surface) | 334.9 |
| | Pd(Surface) | 334.5 |
| **PdO(001)Surface-Bulk-Geometry** | Pd(Bulk) | 335.7 |
| | Pd(Sub-surface) | 335.7 |
| | Pd(Surface) | 336.1 |
| **PdO(001)Surface-Strained** | Pd(Bulk) | 336.8 |

| | | |
|---|---|---|
| | Pd(Sub-surface) | 336.0 |
| | Pd(Surface) | 336.6 |
| **PdO(001)/Pd(100)** | Pd(Bulk) | 334.9 |
| | Pd(Sub-surface) | 335.5 |
| | Pd(Surface) | 336.6 |
| **PdO(001)/Pd(100)-Sub-Surface** | Pd(Bulk) | 334.9 |
| | Pd(Sub-surface) | 335.4 |
| | Pd(Surface) | 335.0 |
| **PdO(001)/Pd(100)-with-$O_v$** | Pd(Bulk) | 334.9 |
| | Pd(Sub-surface) | 335.4 |
| | Pd(Surface) | 336.4 |
| | Pd(Surface-$O_v$) | 335.5 |

Now we move on to the discussion of O 1*s* BEs. The collected O 1*s* spectra at 350 °C in 1 mbar $O_2$ shows a peak at a BE of 529.1 eV due to the lattice oxygen (Figure S13a).(*2, 3*) An additional peak is present at a BE of 530.4 eV previously attributed to the supersaturated adsorbed oxygen layer ($O_{ads}$).(*4*) However, the DFT results in Table S4 suggest this assignment might be incorrect and the oxygen species at BEs above 529.4 eV are more likely related to contaminants. While Table S4 shows such species could also be associated with short lived intermediates such as $CO^*$, $CO_2^*$ or $OCH_3^*$, the concentration of these species is however likely to be below the detection limit. Also note that the measured XPS peaks of the Pd 3$p_{3/2}$ (cyan trace in Figure S13) overlap with the O 1*s* XPS peak. The signal from the gaseous $O_2$ is identified by two peaks at BE of 537.1 eV and 538.2 eV (Figure S13a).(*5, 6*) Increasing the temperature to 550 °C in 1 mbar $O_2$ leads to the presence of an additional component that is 1 eV lower than $O_{lattice}$ (orange line in Figure S13b), in addition to $O_{lattice}$ and $O_{ads}$/$O_{contam}$ peaks discussed above. This component has been assigned previously to surface oxides of Pd,(*5, 6*).This assignment is supported by DFT (Table

S3). The co-feeding of $CH_4$ to the $O_2$ flow ($CH_4$:$O_2$=4.5:1) at 550 °C leads to the disappearance of the $O_{ads}/O_{contam}$ species at a BE of 530.9 eV and almost a complete disappearance of $O_{lattice}$ after introducing $CH_4$ in the $O_2$ flow (Figure S13c), in agreement with the Pd 3d results (Figure 4c). Yet a new peak at a BE of 530.5 eV appears (wine-red line). DFT indicates that the O species at 530.5 eV could be related to bound intermediates (O-bound intermediates) such as CO and $CH_2O$ (Table S4), but again their lifetimes are expected to be short, suggesting the species at 530.5 eV could also be a contaminant that emerges from redox dynamics. The small peaks with BEs higher than Pd 3$p_{3/2}$ indicates the presence of $H_2O$ (BE of 536.4 eV) and $CO_2$ (BE of 535.0 eV) in the gas phase near the sample surface, in agreement with the DFT result that shows when adsorbed, $H_2O$ and $CO_2$ will appear at lower BEs.(*5*-*7*) Consistent with MS data of the *operando* TEM experiment, the O 1*s* XPS reveals the absence of CO near the surface of the Pd catalysts, suggesting the complete oxidation of $CH_4$. Decreasing the temperature from 550 to 350 °C in the $CH_4$/$O_2$ mixture results in the disappearance of the peaks of gas-phase $CO_2$ and $H_2O$ in the O 1*s* XPS (Figure S13d), consistent with lower activity at 350 °C. When the temperature is increased back to 550 °C, the contribution from complete methane oxidation products in the gas-phase returns (Figure S13e).

**Table S4.** O 1*s* core levels calculated using the ΔSCF method. For all the surfaces considered, we have computed the BE of the bulk O atom, a subsurface O atom, and a surface O atom. In addition, for general reference, we have computed the BE of an adsorbed oxygen and oxygens from CO, $CO_2$, OH and $H_2O$ fragments for the Pd(100) surface and OH, $CO_2$ and $CH_2O$ fragments for the PdO(001) surface.

| System | O-atom-Studied | BE (eV) |
|---|---|---|
| **PdO-Bulk** | Bulk-O-Atom | 529.1 |
| **PdO(001)-Surface-PBE-Geometry** | | |
| **i** | Bulk-O-Atom | 529.0 |
| **ii** | Subsurface-O-Atom | 528.7 |
| **iii** | Surface-O-Atom | 526.8 |
| **Pd(100) Surface** | | |
| **i** | O@O adsorbed | 528.2 |
| **ii** | O@CO adsorbed | 530.5 |
| **iii** | O@$CO_2$ adsorbed | 533.4 |
| **Iv** | O@OH adsorbed | 527.6 |
| **v** | O@$H_2O$ adsorbed | 531.8 |
| **PdO(001)-Surface-Strained-Geometry** | | |
| **i** | Bulk-O-Atom | 529.4 |
| **ii** | Subsurface-O-Atom | 528.8 |
| **iii** | Surface-O-Atom | 527.2 |
| **PdO(001)/Pd(100)** | | |
| **i** | Subsurface-O-Atom | 528.4 |
| **ii** | Surface-O-Atom | 527.1 |

| | | |
|---|---|---|
| **iii** | O@OH adsorbed | 528.6 |
| **Iv** | O@$CO_2$ adsorbed | 533.4 |
| **v** | O@$CH_2O$ adsorbed | 530.5 |

## DFT calculations results

### ***Ab initio* atomistic thermodynamics:**

To study the total oxidation of $CH_4$ on the Pd/PdO catalyst, we started by computing the Pd/O surface phase diagram to gain insight into the phases that may participate in the reaction. The stabilities of various surface structures were determined by way of *ab initio* atomistic thermodynamics,(*8*) wherein DFT computed adsorption energies are corrected by the gas-phase chemical potential of $O_2$, $\mu_{O2}(T, p_{O2})$. The surface free energy of a $Pd_xO_y$ overlayer relative to a clean surface can then be written as:

$$\Delta G_{PdxOy}(T, p_{O2}) \approx E_{PdxOy} - x\,\mu_{Pd} - 1/2\,y\,\mu_{O2}(T, p_{O2}) - E_{clean},$$

where $E_{PdxOy}$ and $E_{clean}$ are the DFT total energies of the slab with and without the overlayer of interest, respectively. $\mu_{Pd}$ is the chemical potential of Pd, which we take to be the total energy of the bulk metal. $\mu_{O2}(T, p_{O2})$ is the gas-phase chemical potential of $O_2$ and contains the temperature through tabulated enthalpic and entropic corrections taken from the NIST JANAF tables and an ideal-gas pressure dependence to give:

$$\mu_{O2}(T, p_{O2}) = E_{O2} + H(T, p^0) - H(0, p^0) - TS(T, p^0) + k_B T\, ln(p_{O2}/p^0) \equiv E_{O2} + \tilde{\mu}_{O2}(T, p_{O2}),$$

where $E_{O2}$ is the DFT energy of $O_2$, see below for details of its calculation, and the *H* and *S* terms come from the NIST JANAF tables. Note, in this form, all condensed phases are assumed to have no entropic contribution to the free energy. While this is not true, the difference in the entropic contributions to the

free energies between different overlayers will be small. Thus, the main impact of ignoring the entropic contributions to the free energy of the solid phases is an underestimation of the stability of all phases containing oxygen. We can estimate the magnitude of this error by considering the experimental decomposition temperature of PdO under varying oxygen pressure. Using the PdO heat of formation of 1.17 eV and the decomposition temperatures in literature,(*9, 10*) we can estimate the error in temperature. This results in a temperature error of *ca.* 70 K at *ca.* 1 bar, that is, the prediction from *ab initio* atomistic thermodynamics is 70 K below the observed decomposition temperature for bulk PdO. At an oxygen pressure of *ca.* 0.5 bar the error increases to 76 K, while at *ca.* 0.1 bar the error increases marginally to 77 K. Thus, we expect a temperature error of *ca.* 80 K in the pressure range used in this work. These errors are in line with previous estimates on the Ag/O system.(*11*) The second major source of error in this analysis is the binding energy of the $O_2$ molecule, which is strongly over bound in DFT at the PBE level.(*11*) To avoid this over binding, we took the energy of ½ $O_2$ from the PdO bulk oxide as:

$$1/2\, E_{O2} = E_{PdO} - E_{Pd} - \Delta H_{f,PdO},$$

where $E_{PdO}$ and $E_{Pd}$ are the DFT total energies of bulk PdO and Pd, respectively, and $\Delta H_{f,PdO}$ is the experimental heat of formation of PdO, which we took to be 1.17 eV.(*9*) With these definitions we can compute the surface phase diagram of the Pd/O system as a function of oxygen partial pressure and temperature (Figure S14). Only clean Pd metal, Pd with adsorbed oxygen, and PdO appear on the phase diagram. The oxide overlayers seen in TEM are thus predicted to be present due to kinetic limitations, hence catalyst dynamics. The metastability limits of these overlayers are shown by way of yellow lines in Figure S14.

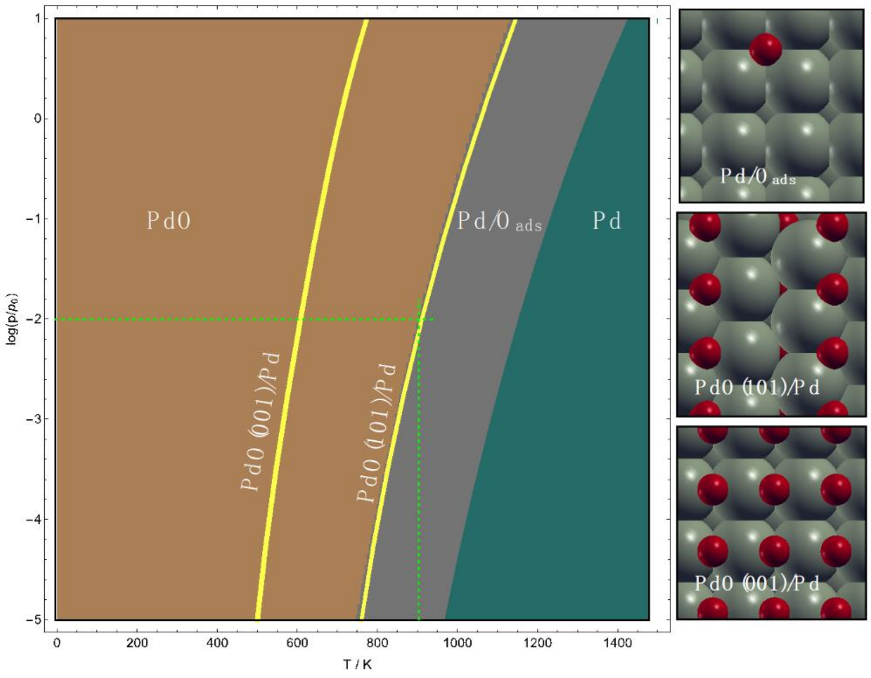


**Figure S14.** Surface phase diagram computed by employing *ab inito* thermodynamics. Only the bulk oxide PdO, metal with adsorbed oxygen, and oxygen free Pd appear as stable phases, as labeled. Yellow lines indicate the areas where the PdO(001)/Pd and PdO(101)/Pd interfaces are stable. The adsorption energies of O adatom per area used for plotting the phase diagram are −0.011, −0.038 and −0.049 eV $(O^* \times Å^2)^{-1}$ for PdO(001)/Pd, PdO(101)/Pd and Pd/$O_{ads}$ respectively. The heat of formation of PdO was taken as −1.17 eV. The right panel shows the top view of PdO(001)/Pd, PdO(101)/Pd and Pd/$O_{ads}$ structures.

**Kinetics of methane oxidation:**

To address the kinetics of the complete oxidation of methane and catalyst dynamics, we considered several structures from the computed phase diagram as possible oxidation catalysts. As Pd metal is observed during *in situ* TEM, we begin our discussion with the metallic phase with a low coverage of oxygen adatoms. To compute the reaction energetics on the clean surface we employed a six layer (3x3) Pd(100) surface and adsorbed $CH_4$ on the surface together with a 1/9 monolayer (ML) coverage of

adsorbed oxygen, see Figure S15. From the figure two hydrogen atoms can be seen to sequentially transfer to adsorbed oxygen to first form a hydroxide and adsorbed $CH_3$, then an adsorbed water molecule and adsorbed $CH_2$. Once the water molecule was created, it was assumed to desorb and was replaced by another oxygen atom to allow the reaction to progress in this low oxygen coverage regime. Two hydrogen atoms are then sequentially transferred from the adsorbed $CH_2$ to the second adsorbed oxygen atom to form a second water molecule and adsorbed carbon. At this point, it should be noted that the remaining carbon atom strongly binds to the four-fold Pd hollow site and hence coke/carbide formation is predicted in this low oxygen coverage limit, though additional oxygen can oxidize the adsorbed carbon to CO, which would desorb in the low oxygen coverage limit, resulting in incomplete methane oxidation under oxygen lean conditions if Pd metal becomes the only available active phase.

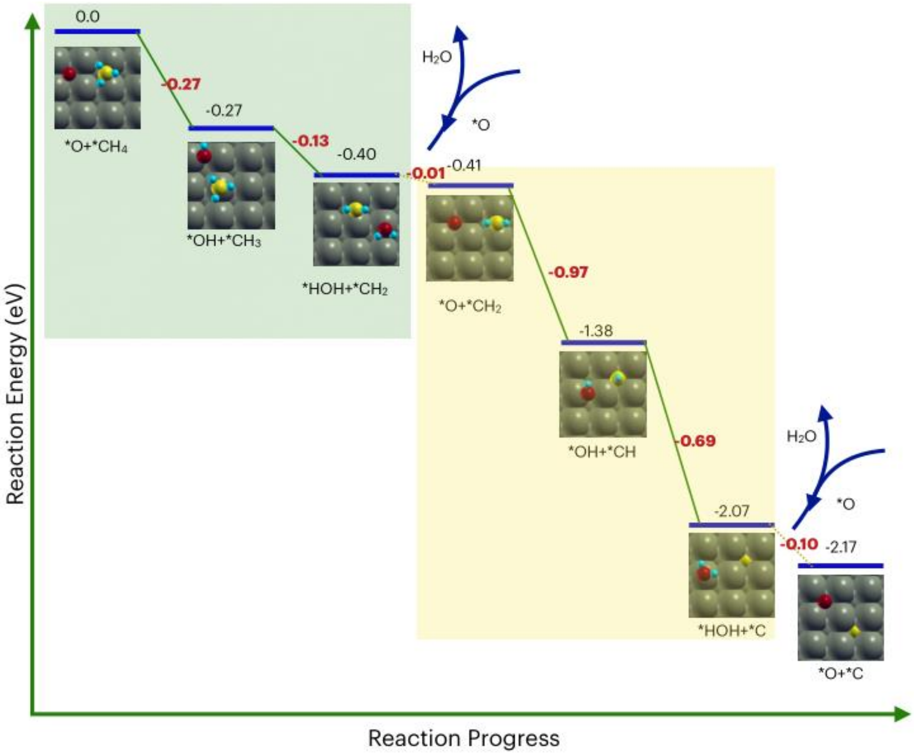


**Figure S15.** Reaction steps involved in methane oxidation on Pd(0) with adsorbed O. Color coding of the atoms are as follows. Dark green (Pd), Red(O), Yellow (C), and Blue (H). Yellow arrows represent the desorption of $H_2O$ from the metal surface.

Closer inspection of Figure S15 reveals the first and second hydrogen transfer reactions are weakly exothermic. Such behavior is consistent with the high gas-phase dissociation enthalpy of the H-$CH_3$ bond(*12*) and the observation that adsorbed O is not effective at promoting C-H bond cleavage on Pd.(*13*) The third and fourth hydrogen transfers are, however, more exothermic. Thus, on the metal surface, the oxygen assisted H transfer from methane follows the gas-phase behavior, with the first steps proving to be the least energetically favorable. This exothermicity will influence the rates of the elementary steps. Usually, metal and metal oxide surfaces follow the Bronsted-Evans-Polyani (BEP) principle, that is, there is a linear relationship between the heat of reaction of an elementary steps and the step's activation energy: $E_a \sim \alpha\, \Delta E_{rxn} + \beta$. This has been shown to hold on bare and oxygen covered Pd metal.(*14*) From the literature,(*15-17*) we can assume the BEP slope ($\alpha$) does not vary considerably across the different hydrogen transfer steps and adopt the universal dehydrogenation BEP relationship: $0.86\, \Delta E_{rxn} + 1.14$ eV,(*16*) which is also close to the universal BEP relationship suggested for C-C, C-O, and O-O coupling and dissociation reactions: $0.84\, \Delta E_{rxn} + 1.92$ eV.(*18*) We have chosen the universal BEP as opposed to a more specific BEP(*14*) to facilitate comparison between the metal and oxide. The assumption of constant BEP slope leads to the conclusion that the steps with highest heats of reaction will have the lowest activation barriers and hence faster reaction kinetics.

On the pristine Pd(100) surface (Figure S15), the early dehydrogenation steps, which are weakly exothermic (-0.27 eV and -0.13 eV, Table S5), will be slower than the final dehydrogenation steps, which are moderately exothermic (-0.97 eV and -0.69 eV). Therefore, the first and second hydrogen transfer will be rate limiting during the initial stages of $CH_4$ oxidation at low oxygen coverage. This behavior is also in line with the BEP relationship derived for methane activation on oxygen covered transition metals.(*14*) Here we estimate a barrier of 0.9-1.0 eV for the two slow steps from the universal BEP, see Table 5. This will make the oxidation reaction slow compared to reoxidation, where the adsorption energy of 2/9 ML O (1.42 eV) gives an estimated (BEP) barrier of 0.7 eV. By way of example, the

Boltzmann factors for the estimated barrier to O-O bond breaking and the first hydrogen transfer step from methane to the surface differ by a factor of 29 at 350 °C and 13 at 550 °C. From the Hertz-Knudsen equation, this implies $p_{CH4}>29p_{O2}$ would be required to maintain the reduced surface at 350 °C, assuming equal sticking probabilities, and $p_{CH4}>13p_{O2}$ would be required at 550 °C. Here the pressures differ by only *ca.* 4.5 and the metallic phase is not maintained.

**Table S5.** Computed heats of reaction and BEP estimated activation energies for methane oxidation on Pd metal. The universal dehydrogenation BEP was employed for the dehydrogenation steps, while the universal BEP C-C, C-O, and O-O coupling/dissociation BEP was used for reoxidation. Note if the later was used for dehydrogenation, the dehydrogenation barriers would increase and not alter the conclusions. [1]The reoxidation reaction was considered to be $O_2 \rightarrow 2O_{ads}$ on the 3x3 Pd(100) surface.

| Step | $E_{rxn}$ / eV | $E_a$ / eV |
|---|---|---|
| **1** | -0.27 | 0.9 |
| **2** | -0.13 | 1.0 |
| **3** | -0.97 | 0.3 |
| **4** | -0.69 | 0.5 |
| **Reoxidation**[1] | -1.42 | 0.7 |

Since our thermodynamic analysis predicts an oxidized surface under the reaction conditions, we can turn our attention to the bulk PdO oxide. Here we considered the PdO(001) surface, where a pure PdO(001) surface was generated using the PBE optimized lattice parameter for the PdO bulk. We found that $CH_4$ oxidation on the PdO surface proceeds through the series of C–H activation steps shown in

Figure 5 in the main text. First, the physiosorbed $CH_4$ undergoes a C–H dissociation to form OH and Pd–$CH_3$ fragments.(*19*) Yet, owing to the known instability of Pd–$CH_3$ species on PdO, a facile migration from Pd to the oxygen site sets in that gives O–$CH_3$ species.(*20*) For brevity, here and below we refer to this dissociation-migration sequence as a single step. (Note, this reaction is qualitatively distinct from that occurring in the low oxygen coverage limit on the Pd(100) surface where the O-C bond formation is inhibited until the $CH_4$ is fully oxidized to adsorbed carbon and water). Subsequently, the C–H activation of the O-$CH_3$ species yields an OH and a O-$CH_2$ fragment that stabilizes as $OCH_2O$ species. This $OCH_2O$ fragment further transfers a hydrogen atom to a surface oxygen to form an OH and a formate-like OCHO species. In the final step, hydrogen is transferred from formate to surface oxygen to yield $CO_2$. Following the previous example of the metal surface, these reaction energies can be used to assess the kinetic stability of the bulk PdO phase by way of the universal BEP scaling relationships.

As discussed above, the first and second C–H activation steps involve hydrogen transfer as well as the O–C bond formation. Therefore, they are strongly exothermic in comparison with the steps found on the metal surface and also with respect to the pure dehydrogenation steps 3 and 4 on the oxide. Assuming the BEP relationship, the third and fourth dehydrogenation step will be rate limiting for methane oxidation over the oxide owing to the small heat of the reaction. To verify the validity of the BEP relationship on the oxide and more accurately quantify the barriers, we determined the activation barriers for the initial four steps of methane oxidation on the unstrained oxide surface using the CI-NEB method, yielding values of 0.67 eV, 0.84 eV, 1.46 eV, and 0.99 eV, respectively. These results are in good agreement with BEP on the assignment of the third step as the rate determining step, but the magnitudes of the computed barriers are higher than those predicted via BEP. With the overall BEP picture supported by the direct simulation, we can identify two interesting differences in methane oxidation over the metal that should be mentioned: i) on the oxide, activation of the H–$CH_3$ bond is no longer rate limiting, making catalysis over the oxide distinct from the gas-phase chemistry of methane or

catalytic oxidation on the metal, and ii) the formation of C–O bonds in kinetically facile steps prevents the incomplete oxidation if the oxide is the active phase because $H_xCO_2$ species is formed before any kinetically slow steps.

We can now estimate the kinetic stability of the PdO phase by comparing the activation energy estimated from the universal BEP relationships for the slow step of dehydrogenation with reoxidation, where the heat of reaction of reoxidation was computed as the total energy associated with filling di-oxygen vacancies on the oxide surface:

$$E_{reoxidation} = E_{Clean} - E_{di-vacancy} - E_{o2},$$

Two divacancy configurations were considered, see Figure S16. The resultant barriers estimated from the universal dehydrogenation BEP and universal BEP for C-C, C-O, and O-O coupling/dissociation are shown in Table S6. Unlike in the case of the metal surface, the estimated activation energies in the case of bulk PdO for the slow methane oxidation step and reoxidation are similar. In fact, the barriers are within the error expected for the BEP fits; the mean absolute error (MAE) of the employed BEP fits are 0.3 eV and 0.35 eV for the dehydrogenation and C-C, C-O, and O-O coupling/dissociation, respectively.(*16, 18*) From the similarity in methane oxidation (surface reduction) and surface reoxidation barriers, we conclude oxidation and reduction of PdO will be competitive. Again, we confirmed the BEP results using CI-NEB. For oxygen dissociation, we found the barrier on the unstrained surface is 2.24 eV, while on the strained surface it is only 1.11 eV. When these are compared to the computed rate determining steps for reduction on the unstrained and strained surface, 1.46 eV and 1.11 eV, we find that reoxidation of the unstrained oxide is more activated than its reduction while on the strained surface, the activation energies of the oxidation and reduction rate limiting steps are nearly identical.

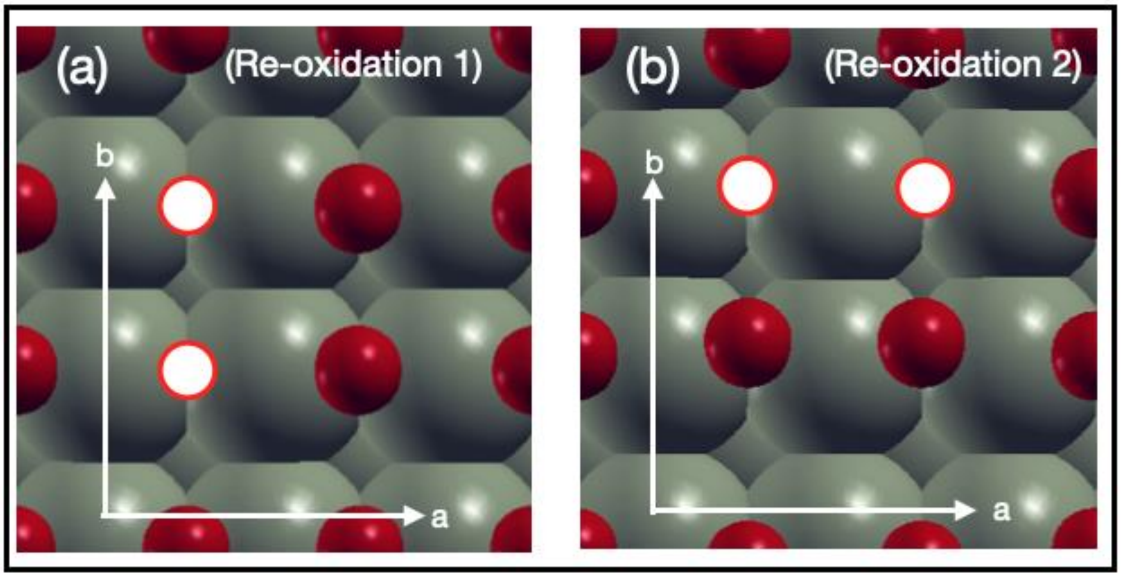


**Figure S16.** Structural model representing two competing reoxidation mechanisms on PdO(001). (a) In Reoxidation 1, the $O_2$ molecule splits and adsorbs onto two different Pd bridge sites shared by four different Pd atoms. (b) While in Reoxidation 2, the $O_2$ molecule splits and adsorbs onto two different bridge sites shared by the same Pd atom.

**Table S6.** Computed heats of reaction for the slow step of methane oxidation over PdO and the reoxidation of $PdO_x$ together with the barriers estimated using the universal dehydrogenation BEP and universal BEP for C-C, C-O, and O-O coupling/dissociation. The former was used for the dehydrogenation step and the later for the reoxidation step.

| Step | $E_{rxn}$ / eV | $E_a$ / eV |
|---|---|---|
| **Dehydrogenation step 4** | -1.12 | 0.2 |
| **Reoxidation 1 (vacancy configuration 1)** | -1.52 | 0.6 |
| **Reoxidation 2 (vacancy configuration 2)** | -2.00 | 0.2 |

As catalyst reoxidation and reduction through methane oxidation are competitive, catalyst dynamics emerge. It is thus important to also consider the Pd/PdO interface. Here two cases can be considered: 1) multilayer PdO and 2) single layer PdO.

When multilayer PdO domains are in contact with Pd metal, the oxide is strained into epitaxy with the metal at the metal/oxide interface, as shown in Figure 3, where the amount of strain depends on the distance to the interface. When a PdO monolayer is present a Pd(200)/PdO(002) interface appears to form and maintain an epitaxial relationship with the metal. (Note this phase was observed at 550 °C. Figure 3k shows a PdO(100) overlayer on Pd(111)). To study the catalytic behavior of these structures we modelled a PdO(001) overlayer on a Pd(100) surface, which we denote Pd/PdO. The Pd/PdO surface is modelled by placing a PdO(001) overlayer on the Pd(100) surface while keeping the lattice constant of Pd(100) fixed. This makes the overlayer PdO surface strained with respect to the bulk PdO lattice parameter. The strained bulk oxide was modeled using the same in-plane lattice parameters as the Pd/PdO but the model included effectively 2 layers of PdO rather than PdO and metal.

We found both the strained PdO and Pd/PdO surfaces oxidize methane through the same five-step mechanism as unstrained PdO. However, the strain increases the exothermicity of the slow dehydrogenation steps at the expense of the exothermicity of the early fast dehydrogenation steps, see Figure 5. Overall, this equating of heats of reaction for each elementary step is expected to increase the rate of methane oxidation. Estimating the barriers of these slow steps using the universal dehydrogenation BEP relationship suggests the reactions even become barrierless while the reaction energies for two reoxidation steps involving different di-oxygen vacancy configurations are shown in Table S7. The estimated activation energies for these steps are 0.8 eV on the Pd/PdO interface and 0.6 eV on the strained PdO. In Table S8 we have summarized the activation energies of all the catalytic intermediates on the unstrained, strained and overlayer surfaces estimated using CI-NEB. The barriers computed using CI-NEB show that, unlike the case of Pd metal, the third dehydrogenations step remains

rate-determining. However, the barriers decrease in the case of stained PdO as suggested by the BEP relations. Overall, this will drive small PdO domains to metallic Pd. Thermodynamics will, however, push the catalyst back to the PdO, thereby setting up catalyst dynamics, the kinetics of which will show strong size dependence owing to the influence of interfacial Pd/PdO strain.

**Table S7.** The reaction energies for two competing reoxidation step, see Figure S16.

| Step | (Pd/PdO): $E_{rxn}$ / eV; $E_a$ / eV | (Strained PdO): $E_{rxn}$ / Ev; $E_a$ / eV |
|---|---|---|
| **Reoxidation 1 (vacancy configuration 1)** | -1.36 ; 0.8 | -1.57; 0.6 |
| **Reoxidation 2 (vacancy configuration 2)** | -1.28; 0.8 | -1.61; 0.6 |

**Table S8.** Activation energies of all strained, unstrained and over layer structures estimated using CI-NEB. For the overlayer we only computed the activation energies for steps 3 and 4 as the BEP results suggest these are rate limiting. *In this case the error on the climbing image is still above 0.05 eV/A, and hence is not well converged.

| Structure | $E_a$ (eV) |
|---|---|
| **Bulk PdO Surface** | |
| **Step 1** | 0.63 |
| **Step 2** | 0.84 |
| **Step 3** | 1.46 |
| **Step 4** | 0.99 |
| **$O_2$ dissociation** | 2.24 |
| **Strained PdO Surface** | |
| **Step 1** | 0.68 |

| | |
|---|---|
| **Step 2** | 0.73* |
| **Step 3** | 1.12 |
| **Step 4** | 0.83 |
| **$O_2$ dissociation** | 1.11 |
| **Pd/PdO overlayer surface** | |
| **Step 3** | 0.98 |
| **Step 4** | 0.93 |

**Microkinetic model:**

Phase oscillations will not necessarily emerge in reactive systems with similar activation energies like that investigated in this work. Steady-state solutions with constant coverages are also possible. Thus, to verify that the DFT results are consistent with phase oscillations, we parametrized a simple microkinetic model using the activation energies computed on the strained PdO surface to show how dynamics can emerge. This model aims to qualitatively capture the observed dynamic behavior by including the phase transition between Pd and PdO and the slow chemical steps associated with methane oxidation. We consider the following steps:

$$\mathrm{O}_{2,\mathrm{gas}} + * \rightarrow 2\mathrm{O}_{\mathrm{ads}}, \tag{R1}$$

$$\mathrm{CH}_{4,\mathrm{gas}} + *_{\mathrm{oxide}} \rightleftarrows \mathrm{CH}_{4,\mathrm{ads}}, \tag{R2}$$

$$\mathrm{CH}_{4,\mathrm{ads}} + 2\mathrm{O}_{\mathrm{ads}} \rightarrow \mathrm{I}_{\mathrm{ads}}, \tag{R3}$$

$$\mathrm{I}_{\mathrm{ads}} + 2\mathrm{O}_{\mathrm{ads}} \rightarrow \mathrm{CO}_{2,\mathrm{gas}} + 2\mathrm{H}_2\mathrm{O}, \tag{R4}$$

and

$$\mathrm{O}_{\mathrm{ads}} \leftrightarrow \mathrm{oxide}. \tag{R5}$$

We consider that $O_2$ dissociates when adsorbing on the surface via R1, which is taken as irreversible because we are only concerned with conditions far below the PdO decomposition temperature. We assume $CH_4$ adsorbs reversibly on the oxide surface since DFT predicts physorption is the first step in the reaction. Adsorbed methane can then react with two adsorbed oxygen atoms as shown in the main text to produce an adsorbed intermediate via R3. This reaction is slow compared to the quasi-equilibrated adsorption of methane but fast compared to further oxidation of the intermediate via step R4, the rate of which is taken to be determined by the RDS for methane oxidation. Note that the oxide phase formed via R5 is required for methane sticking and eventual oxidation. The measured activation energy for this step (1.5 eV) is higher than all computed activation energies.(*2*)

The corresponding kinetic equations are:

$$\frac{\mathrm{d}\theta_{\mathrm{O_{ads}}}}{\mathrm{dt}} = k_1\left(1-\theta_{\mathrm{CH_{4,ads}}}-\theta_{\mathrm{O_{ads}}}-\theta_{\mathrm{I_{ads}}}\right)^2-2k_3\left(\theta_{\mathrm{CH_{4,ads}}}\theta_{\mathrm{O_{ads}}}{}^2\right)-2k_4\left(\theta_{\mathrm{I_{ads}}}\theta_{\mathrm{O_{ads}}}{}^2\right), \quad \text{(R6)}$$

$$\frac{\mathrm{D}\theta_{\mathrm{CH_{4,ads}}}}{\mathrm{dt}} = k_2\left(s_{\mathrm{oxide}}\left(1-\theta_{\mathrm{CH_{4,ads}}}-\theta_{\mathrm{I_{ads}}}\right)\right)-k_3\left(\theta_{\mathrm{CH_{4,ads}}}\theta_{\mathrm{O_{ads}}}{}^2\right), \quad \text{(R7)}$$

$$\frac{\mathrm{d}\theta_{\mathrm{I_{ads}}}}{\mathrm{dt}} = k_3\left(\theta_{\mathrm{CH_{4,ads}}}\theta_{\mathrm{O_{ads}}}{}^2\right)-k_4\left(\theta_{\mathrm{I_{ads}}}\theta_{\mathrm{O_{ads}}}{}^2\right), \quad \text{(R8)}$$

and

$$\frac{\mathrm{d}\theta_{\mathrm{oxide}}}{\mathrm{dt}} = k_5\left(f\left(\theta_{\mathrm{O_{ads}}}\right)-\theta_{\mathrm{oxide}}\right). \quad \text{(R9)}$$

In R6 $k_1$ is the rate constant for dissociative $O_2$ adsorption, which we take to be the Hertz Knudsen flux multiplied by a probability to activate $O_2$. In R7 $k_2$ is the Hertz Knudsen flux, and $s_{\mathrm{oxide}}$ is the sticking coefficient on oxide, which we take to be unity. Although R8 is not an elementary step, $k_3$ is taken as the rate constant for the first step in activating methane on the oxide to form an intermediate, where methane forms a C-O bond and transfers H to a second O, hence two O atoms are involved in the reaction. Then $k_4$ is the rate of consumption of this intermediate through the RDS, which also consumes

two additional oxygen atoms. While this is a non-elementary step, we assume the apparent activation energy is identical to the computed activation energy of the RDS. In R9 adsorbed oxygen precipitates oxide in a non-elementary reaction with rate constant $k_5$. Here the function $f(\theta_{O_{ads}})$ is a step function that turns on oxide formation once the oxygen coverage exceeds 25%. The activation energy associated with this transformation is taken as 1.5 eV, from Ref. 2. Using this with the DFT computed activation energies and apparent prefactors of $10^8$ for all steps expect oxide formation, where we used $10^9$ owing to the high measured apparent prefactor,(*2*) we can compute the oxide coverage as a function of time on stream, where we chose the experimental conditions of 29.5 mbar $CH_4$ and 8.8 mbar $O_2$ at 550 C. The result is shown in Figure S17, where the oxide coverage is plotted against time. Here we start with a clean surface and after an initial transient period, stable phase oscillations emerge. The appearance and rate of the phase oscillations are intimately tied to the metal/oxide phase transition. Specifically, the phase oscillations emerge because the metal/oxide phase transition introduces a slow time scale resulting in oscillations occurring on the seconds timescale.

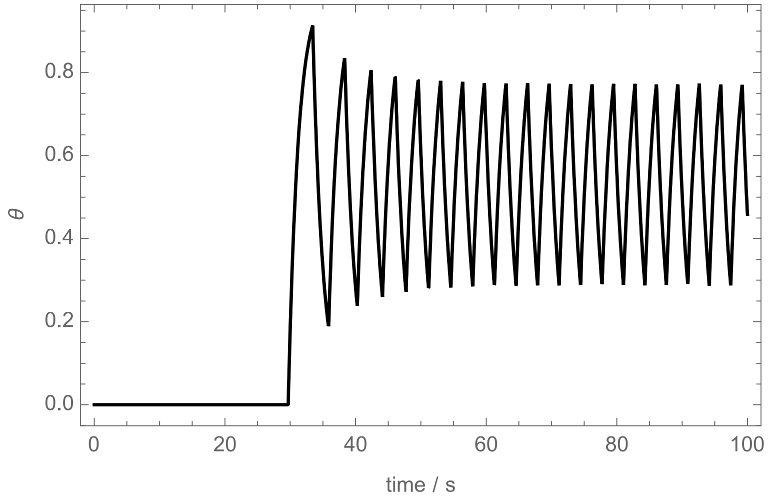


**Figure S17.** Oxide coverage vs time found using the DFT parameterized microkinetic model under 29.5 mbar $CH_4$ and 8.8 mbar $O_2$ at 550 C.

Supplementary References


1. W. E. W. NIST Mass Spectrometry Data Center, director, "Mass Spectra" in NIST Chemistry WebBook, NIST Standard Reference Database Number 69, Eds. P.J. Linstrom and W.G. Mallard, National Institute of Standards and Technology, Gaithersburg MD, 20899, https://doi.org/10.18434/T4D303, (retrieved August 6, 2022)
2. G. Ketteler *et al.*, In situ spectroscopic study of the oxidation and reduction of Pd(111). *J. Am. Chem. Soc.* **127**, 18269-18273 (2005).
3. H. Gabasch *et al.*, In situ XPS study of Pd(111) oxidation at elevated pressure, Part 2: Palladium oxidation in the $10^{-1}$mbar range. *Surf. Sci.* **600**, 2980-2989 (2006).
4. D. Zemlyanov *et al.*, In situ XPS study of Pd(111) oxidation. Part 1: 2D oxide formation in $10^{-3}$mbar $O_2$. *Surf. Sci.* **600**, 983-994 (2006).
5. S. Blomberg *et al.*, In situ X-ray photoelectron spectroscopy of model catalysts: at the edge of the gap. *Phys. Rev. Lett.* **110**, 117601 (2013).
6. V. R. Fernandes *et al.*, Reversed hysteresis during CO oxidation over $Pd_{75}Ag_{25}$(100). *ACS Catal.* **6**, 4154-4161 (2016).
7. I. P. Prosvirin, A. V. Bukhtiyarov, H. Bluhm, V. I. Bukhtiyarov, Application of near ambient pressure gas-phase X-ray photoelectron spectroscopy to the investigation of catalytic properties of copper in methanol oxidation. *Appl. Surf. Sci.* **363**, 303-309 (2016).
8. K. Reuter, M. Scheffler, Composition, structure, and stability of $RuO_2$(110) as a function of oxygen pressure. *Phys. Rev. B* **65**, 035406 (2001).
9. N. N. Smirnova, T. A. Bykova, N. A. Polotnyanko, N. D. Shikina, I. L. Khodakovskii, Low-temperature heat capacity and standard entropy of PdO(cr.). *Russ. J. Phys. Chem. A* **84**, 1851-1855 (2010).
10. H. Zhang, J. Gromek, G. W. Fernando, H. L. Marcus, S. Boorse, PdO/Pd system equilibrium phase diagram under a gas mixture of oxygen and nitrogen. *J. Phase Equilib.* **23**, 246 (2002).
11. T. E. Jones *et al.*, Thermodynamic and spectroscopic properties of oxygen on silver under an oxygen atmosphere. *Phys. Chem. Chem. Phys.* **17**, 9288-9312 (2015).
12. D. Ciuparu, M. R. Lyubovsky, E. Altman, L. D. Pfefferle, A. Datye, Catalytic combustion of methane over palladium-based catalysts. *Catal. Rev.* **44**, 593-649 (2002).
13. D. Hibbitts, M. Neurock, Promotional effects of chemisorbed oxygen and hydroxide in the activation of C–H and O–H bonds over transition metal surfaces. *Surf. Sci.* **650**, 210-220 (2016).
14. B. Xing, X.-Y. Pang, G.-C. Wang, C–H bond activation of methane on clean and oxygen pre-covered metals: A systematic theoretical study. *J. Catal.* **282**, 74-82 (2011).
15. J. Cheng *et al.*, Brønsted−Evans−Polanyi relation of multistep reactions and volcano curve in heterogeneous catalysis. *J. Phys. Chem. C* **112**, 1308-1311 (2008).
16. S. Wang *et al.*, Universal transition state scaling relations for (de)hydrogenation over transition metals. *Phys. Chem. Chem. Phys.* **13**, 20760-20765 (2011).
17. J. E. Sutton, D. G. Vlachos, A theoretical and computational analysis of linear free energy relations for the estimation of activation energies. *ACS Catal.* **2**, 1624-1634 (2012).
18. S. Wang *et al.*, Universal Brønsted-Evans-Polanyi relations for C–C, C–O, C–N, N–O, N–N, and O–O dissociation reactions. *Catal. Lett.* **141**, 370-373 (2011).
19. C. Coperet, C−H bond activation and organometallic intermediates on isolated metal centers on oxide surfaces. *Chem. Rev.* **110**, 656-680 (2010).
20. M. Blanco-Rey, S. J. Jenkins, Methane dissociation and methyl diffusion on PdO{100}. *J. Chem. Phys.* **130**, 014705 (2009).
