# Supplementary material for: Redox dynamics and surface structures of an active palladium catalyst during methane oxidation
Source: Nat Commun. 2024 Jun 1;15:4678. doi: 10.1038/s41467-024-49134-y (PMC11144237; doi:10.1038/s41467-024-49134-y)
Supplement: Supplementary file 3 — Description of Additional Supplementary Information [file 41467_2024_49134_MOESM3_ESM.pdf]

Description of Additional Supplementary Files

Supplementary Movie 1: Morphological and structural changes of Pd particles during increasing temperature from 350 °C to 800 °C in a gas mixture of CH_4 and O_2.

Supplementary Movie 2: Morphological and structural changes of Pd particles during decreasing temperature from 800 °C to 550 °C in a gas mixture of CH_4 and O_2.

Supplementary Movie 3: Particle fragmentation during addition of CH_4 into the O_2 flow at 550 °C.

Supplementary Movie 4: Particle dynamics observed at a low magnification at 350 °C.

Supplementary Movie 5: Particle dynamics observed at a low magnification at 550 °C.

Supplementary Movie 6: In situ electron diffraction of Pd particles at 350 °C.

Supplementary Movie 7: In situ electron diffraction of Pd particles at 550 °C.

Supplementary Movie 8: Particle dynamics observed at a medium magnification at 550 °C.

Supplementary Movie 9: Oscillatory phase transition between Pd and PdO on the metallic surface.

Supplementary Movie 10: Oscillatory phase transition between Pd and PdO on the oxide surface.
