# Supplementary material for: Redox dynamics and surface structures of an active palladium catalyst during methane oxidation
Source: Nat Commun. 2024 Jun 1;15:4678. doi: 10.1038/s41467-024-49134-y (PMC11144237; doi:10.1038/s41467-024-49134-y)
Supplement: Supplementary file 14 — Reporting Summary [file 41467_2024_49134_MOESM14_ESM.pdf]

nature portfolio

Corresponding author(s): Travis Jones, Xing Huang

Last updated by author(s): May 1, 2024

Reporting Summary

Nature Portfolio wishes to improve the reproducibility of the work that we publish. This form provides structure for consistency and transparency in reporting. For further information on Nature Portfolio policies, see our Editorial Policies and the Editorial Policy Checklist.

Statistics

For all statistical analyses, confirm that the following items are present in the figure legend, table legend, main text, or Methods section.

n/a	Confirmed	
☒	☐	The exact sample size (*n*) for each experimental group/condition, given as a discrete number and unit of measurement
☒	☐	A statement on whether measurements were taken from distinct samples or whether the same sample was measured repeatedly
☒	☐	The statistical test(s) used AND whether they are one- or two-sided<br>*Only common tests should be described solely by name; describe more complex techniques in the Methods section.*
☒	☐	A description of all covariates tested
☒	☐	A description of any assumptions or corrections, such as tests of normality and adjustment for multiple comparisons
☒	☐	A full description of the statistical parameters including central tendency (e.g. means) or other basic estimates (e.g. regression coefficient) AND variation (e.g. standard deviation) or associated estimates of uncertainty (e.g. confidence intervals)
☒	☐	For null hypothesis testing, the test statistic (e.g. *F*, *t*, *r*) with confidence intervals, effect sizes, degrees of freedom and *P* value noted<br>*Give P values as exact values whenever suitable.*
☒	☐	For Bayesian analysis, information on the choice of priors and Markov chain Monte Carlo settings
☒	☐	For hierarchical and complex designs, identification of the appropriate level for tests and full reporting of outcomes
☒	☐	Estimates of effect sizes (e.g. Cohen's *d*, Pearson's *r*), indicating how they were calculated

Our web collection on statistics for biologists contains articles on many of the points above.

Software and code

Policy information about availability of computer code

Data collection	Gatan Microscopy Suite (version 3.30.2016.0) was used to collect images during in situ TEM experiments. The QuantumESPRESSO (version 6.7) package with the GGA-PBE exchange and correlation potential was employed to perform DFT calculations. SES software (version 1.9.5) was used to collect the XPS data.
Data analysis	Digital Micrograph (version 3.21.1374.0) and ImageJ (version 1.52a) were used to analyze the TEM images. CasaXPS software (version 2.3.17PR1.1) was used to analyze the XPS data. All the crystal structures obtained from DFT calculations were plotted using XCrySDen(version 1.6.2). The thermodynamic phase diagram was plotted using Mathematica (version 12).

For manuscripts utilizing custom algorithms or software that are central to the research but not yet described in published literature, software must be made available to editors and reviewers. We strongly encourage code deposition in a community repository (e.g. GitHub). See the Nature Portfolio guidelines for submitting code & software for further information.

Data

Policy information about availability of data

All manuscripts must include a data availability statement. This statement should provide the following information, where applicable:

- Accession codes, unique identifiers, or web links for publicly available datasets
- A description of any restrictions on data availability
- For clinical datasets or third party data, please ensure that the statement adheres to our policy

The data that support the findings of this study are included in the published article and its supplementary information files. These data are also available from the corresponding authors upon reasonable request.

Research involving human participants, their data, or biological material

Policy information about studies with human participants or human data. See also policy information about sex, gender (identity/presentation), and sexual orientation and race, ethnicity and racism.

Reporting on sex and gender	Does not apply
Reporting on race, ethnicity, or other socially relevant groupings	Does not apply
Population characteristics	Does not apply
Recruitment	Does not apply
Ethics oversight	Does not apply

Note that full information on the approval of the study protocol must also be provided in the manuscript.

Field-specific reporting

Please select the one below that is the best fit for your research. If you are not sure, read the appropriate sections before making your selection.

☐ Life sciences ☐ Behavioural & social sciences ☒ Ecological, evolutionary & environmental sciences

For a reference copy of the document with all sections, see nature.com/documents/nr-reporting-summary-flat.pdf

Ecological, evolutionary & environmental sciences study design

All studies must disclose on these points even when the disclosure is negative.

Study description	Does not apply
Research sample	Does not apply
Sampling strategy	Does not apply
Data collection	In situ TEM data were collected by Xing Huang using a double-corrected TEM (GrandARM 300F) and a DENSsolutions in situ gas holder (connected to a gas feeding system). In situ MS data were recorded by Masahiro Hashimoto and Xing Huang using JEOL JMS-Q1500GC mass spectrometer. DFT calculations were performed by Praveen C. S. and Travis Jones using the Quantum ESPRESSO (version 6.7) package with the GGA-PBE exchange and correlation potential using projected augmented wave pseudopotentials taken from the PSlibrary, with a kinetic energy (charge density) cutoff of 55 Ry (600 Ry). The NAP-XPS data were collected by Alexander Klyushin at the HIPPIE beamline of MAX IV Laboratory. Details on how the data were recorded can be found in the Methods section of the manuscript.
Timing and spatial scale	The data collection spanned from April 2018 to April 2024. In detail, in situ TEM data were collected from April 2018 to June 2020, while DFT calculations were conducted from June 2021 to April 2024.
Data exclusions	No data were excluded from the analysis.
Reproducibility	In situ TEM experiments were repeated multiple times to verify the reproducibility, and they yielded similar results.
Randomization	Our study did not involve sample allocation or randomization as we focused on a specific sample (Pd nanopowder). Our objective was to investigate the dynamic behavior of this sample under catalytic reaction conditions.

Blinding | Does not apply

Did the study involve field work? ☐ Yes ☒ No

Reporting for specific materials, systems and methods

We require information from authors about some types of materials, experimental systems and methods used in many studies. Here, indicate whether each material, system or method listed is relevant to your study. If you are not sure if a list item applies to your research, read the appropriate section before selecting a response.

Materials & experimental systems

n/a	Involved in the study
☒	☐ Antibodies
☒	☐ Eukaryotic cell lines
☒	☐ Palaeontology and archaeology
☒	☐ Animals and other organisms
☒	☐ Clinical data
☒	☐ Dual use research of concern
☒	☐ Plants

Methods

n/a	Involved in the study
☒	☐ ChIP-seq
☒	☐ Flow cytometry
☒	☐ MRI-based neuroimaging

Plants

Seed stocks | Does not apply

Novel plant genotypes | Does not apply

Authentication | Does not apply
